# First Principles Study on the Electronic Properties of Zn_64_Sb_64−_*_x_*Te*_x_* Solid Solution (*x* = 0, 2, 3, 4)

**DOI:** 10.3390/ijms12053162

**Published:** 2011-05-13

**Authors:** Jian-Hua Zhao, Er-Jing Han, Tian-Mo Liu, Wen Zeng

**Affiliations:** 1 College of Materials Science and Engineering, Chongqing University, Chongqing 400030, China; E-Mails: erjing_4630@yahoo.com.cn (E.-J.H.); tmliu@cqu.edu.cn (T.-M.L.); zeng_wen1982@yaoo.com.cn (W.Z.); 2 National Engineering Research Center for Magnesium Alloys, Chongqing University, Chongqing 400030, China

**Keywords:** first-principles, ZnSb, electronic structure, *n*-type

## Abstract

The electronic properties of Te doped-ZnSb systems are investigated by first-principles calculations. We focus on the Zn_64_Sb_64−_*_x_*Te*_x_* systems (*x* = 0, 2, 3, 4), which respond to the 0, 1.56at%, 2.34at% and 3.12at% of Te doping concentration. We confirm that the amount of Te doping will change the conductivity type of ZnSb. In the cases of *x* = 2 and 3, we find that the Te element in ZnSb introduces some bands originating from Te s and p orbits and a donor energy level in the bottom of the conduction band, which induce the *n*-type conductivity of ZnSb. From these findings for the electronic structure and the conductivity mechanism, we predict that Te doping amounts such as 1.56at% and 2.34at% can be considered as suitable candidates for use as donor dopant.

## 1. Introduction

ZnSb is one of the stable compounds used in intermediate temperatures, and has attracted a lot of interest as a thermoelectric material due to its low thermal conductivity [1–3]. Many measures have been taken in an attempt to improve the thermoelectric figure of merit, such as the investigations of fabrication and doping or solid solution of foreign elements [4–6]. Furthermore, a thermoelectric module can generally be constructed using a unicouple of *p*- and *n*-type semiconductors. Interestingly, it is found that doping a proper amount of Te resulted in a change of ZnSb conductivity type from *p* to *n* [7]. Te doping in ZnSb may alter the Zn/Sb ratio of the bulk material and provide additional scattering mechanism. This doping changes the electronic structure of the material and offers opportunities for optimizing its thermoelectric performance.

However, the current understanding of the doping effect on the ZnSb is based mainly on experimental studies via a trial-and-error design [8]. The mechanism behind this phenomenon is still unclear and there is a lack of detail to understand it. In this respect, it is crucial to know how to choose the proper concentration of dopant accurately. First principles simulation may be an effective method to understand the mechanism of the experimental study [9–11]. Surprisingly, there have been few theoretical studies of the effect of dopant on the properties of ZnSb, especially from an electronic point of view [12,13].

In this work, we investigate atomic and electronic structures of the Zn_64_Sb_64−_*_x_*Te*_x_* systems from first-principles calculations. In particular, we focus on the four models as Zn_64_Sb_64_, Zn_64_Sb_62_Te_2_, Zn_64_Sb_61_Te_3_ and Zn_64_Sb_60_Te_4_, which represent the doing amount of Te as 0, 1.56at%, 2.34at% and 3.12at% respectively. The main objective of this study is to understand the doping effect on the electronic structure of ZnSb from first principles calculations and provide insight into how to find a proper doping concentration, which makes the ZnSb exhibit special electronic properties.

## 2. Calculation Models and Methods

**Figure 1.**
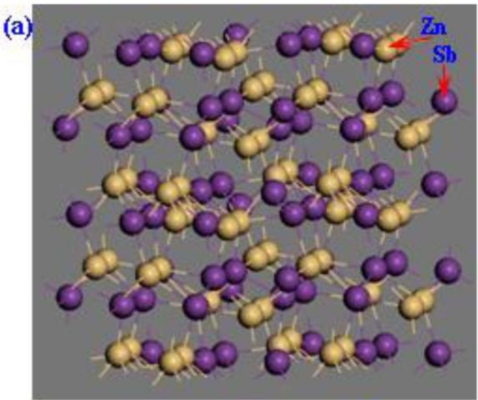

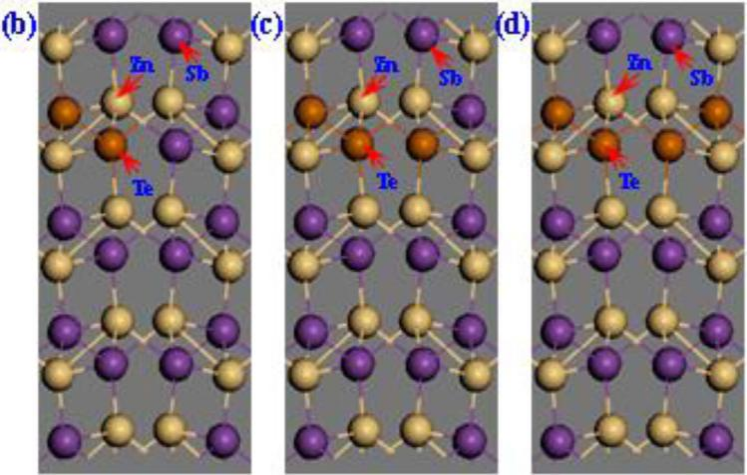
Supercell model of (**a**) Zn_64_Sb_64_; (**b**) Zn_64_Sb_62_Te_2_-S1; (**c**) Zn_64_Sb_61_Te_3_-S2 and (**d**) Zn_64_Sb_60_Te_4_-S3.

It is well known that ZnSb belongs to the orthorhombic symmetry D2h space group P/bca [14]. Based on the optimized structure of perfect ZnSb, the supercell containing 128 atoms was established under periodic boundary conditions by repeating the unit cell 2 × 2 × 2 along the a, b, c directions as shown in Figure 1a. To investigate the doping effect, we doped different amounts of substitutional metallic atoms (Te) into the ZnSb from consideration of symmetry and confirmed that this doping method made the system the most energetically stable. We have constructed a total of three possible models, including Zn_64_Sb_62_Te_2_ (S1), Zn_64_Sb_61_Te_3_ (S2) and Zn_64_Sb_60_Te_4_ (S3), respectively, as illustrated in Figure 1b, c and d.

We performed the first-principles calculations using the VASP code within the density functional theory (DFT) framework [15]. The structure properties of ZnSb systems were studied by using the Perdew–Burke–Ernzerhof (PBE) functional form of the generalized gradient approximation (GGA) [16] for the exchange-correlation potential and ultrasoft pseudopotentials [17]. A cut off energy of 450 eV and a regular Monkhorst-Pask grid of 4 × 4 × 4 *k* points were adopted to ensure energy convergence to less than 1–2 meV/atom. The following states were treated as valence states: Zn (3p^6^3d^10^4s^2^), Sb (5s^2^5p^3^), Te (5s^2^5p^4^).

## 3. Results and Discussion

Firstly, the pure ZnSb system is studied for comparison. The supercell mentioned above has been optimized and then the lattice parameters (a, b and c) of unit cell have been obtained, as shown in Table 1.

As can be seen, the calculated lattice parameters are in good agreement with the experimental data [18], which allows us to assume the reliability of our model. We relaxed the Zn_64_Sb_62_Te_2_, Zn_64_Sb_61_Te_3_ and Zn_64_Sb_60_Te_4_ structures, respectively. In the three cases, the structure relaxation shows the same tendency with each atom. A careful comparison of the displacement of each atom along the [001] direction with respect to the equivalent position, shows that the Te, Zn, and Sb atoms deform somewhat (the relaxation distant for each atom is less than 0.04 Å, 0.02 Å, 0.01 Å). It is indicated that the Te doping does not induce other structural modifications.

**Figure 2.**
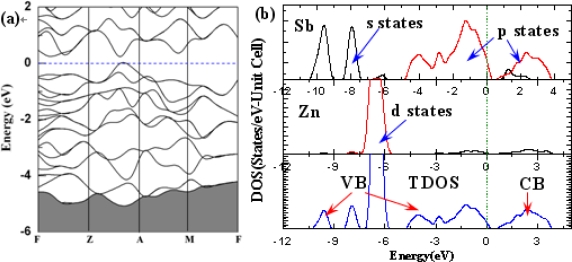
(**a**) The calculated band structure; (**b**) Total and partial densities of states of Zn_64_Sb_64_. The Fermi level (*E_F_*) is set as relative zero.

Figure 2 shows the band structure and density of states (DOS) of a Zn_64_Sb_64_ cluster. A simple glance at Figure 2a shows that the overall shapes of band structures and calculated energy band gap is 0.22 eV, which is smaller than the experimental data [19], but close to the calculated value of 0.2 eV [13,20]. The deviation from the experimental value can be attributed to the well-known drawback of DFT, but the results are also advisable for the qualitative analysis [21]. Figure 2b shows the TDOS of ZnSb and partial density of states (PDOS) of Zn and Sb atoms. One can see that the valence band (VB) can generally be divided into two regions, the lower VB within −11 eV to −5 eV and the upper valence band within −5 eV to 0. The upper VB is mainly contributed by Sb p states, and the lower VB is chiefly contributed by the Sb s states and Zn d states, the conduction band (CB) is primarily contributed by Sb s and p states. We noted that the contribution of Zn based states to the valence band is substantial, which is anticipated from the small electronegativity difference between Zn and Sb. So, the ZnSb can be considered as a polarized but covalently bonded framework structure [12].

**Figure 3.**
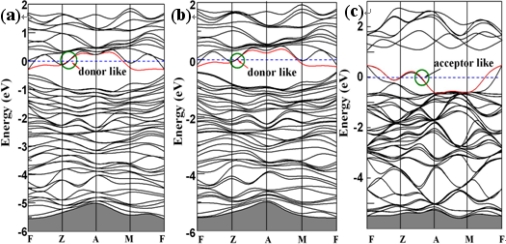
Band structure of (**a**) Zn_64_Sb_62_Te_2_ (model S1); (**b**) Zn_64_Sb_61_Te_3_ (model S2) and (**c**) Zn_64_Sb_60_Te_4_ (model S3).

To gain insight into the electronic properties of ZnSb solution after Te doping, we first present band structures for the three models in Figure 3. One feature in common is that there does not appear to be a gap state in any case, which indicates that ZnSb remains its semiconducting nature after doping Te. It is noteworthy that some new bands appear around the Femi level (*E_F_*) for different cases. In the cases of S1 and S2 (Figure 3a,b), the CB are remarkably reduced in the band-gap region, and some new donor-like levels appear near the *E_F_*, which can contribute to the *n*-type conductor, while in S3 case (Figure 3c), some acceptor-like levels appear near the top of VB, which may induce the *p*-type conductive for ZnSb. These indicate that the different doping concentration in ZnSb will lead to the conductivity transfer between *p*-type and *n*-type.

To further understand the behavior of the electronic structure for the ZnSb systems, the TDOS and PDOS of each atom have also been investigated.

**Figure 4.**
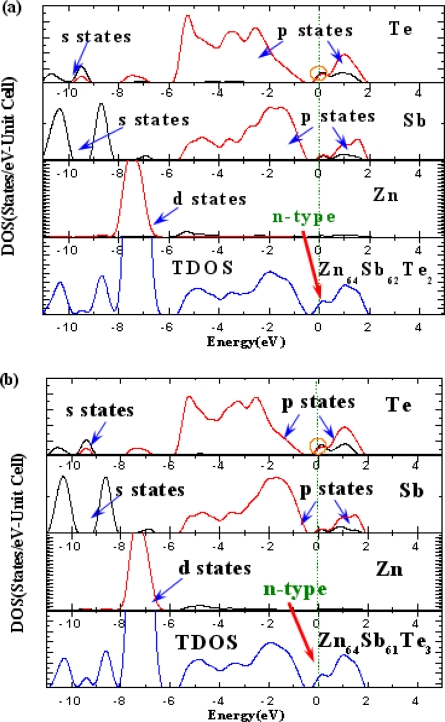

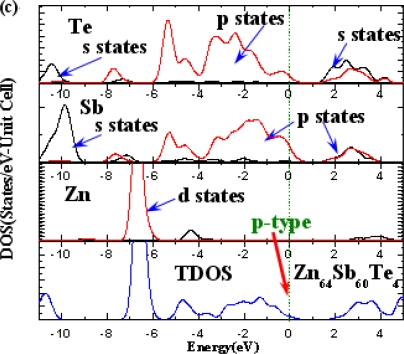
Total and partial densities of states of (**a**) Zn_64_Sb_62_Te_2_ (model S1); (**b**) Zn_64_Sb_61_Te_3_ (model S2) and (**c**) Zn_64_Sb_60_Te_4_ (model S3).

As illustrated in Figure 4, we see that they share some features: No surface states appear between the band gaps, the VB can generally be divided into two regions, the lower VB within −11 eV to −5.6 eV, and the upper VB within −5.4 eV to 0. The lower VB is mainly contributed by Zn d states, as compared to DOS of ZnSb (Figure 2b), lower VB change is somewhat slight, which means Te influences slightly the lower VB of ZnSb.

However, the electronic structure near the *E_F_* level is altered significantly via impurity doping, as weak states appear at *E_F_*, which means that the dope system is somewhat metalized. Two notable cases in S1 and S2 systems are as shown in Figure 4a and b. In addition, one can see the donor-like across the *E_F_*, which makes the ZnSb exhibit *n*-type conductivity. A careful PDOS analysis reveals that the *n*-type behavior is primarily due to s and p states of the Te atom, which consequently provides a weak but visible peak as the donors. Moreover, one can see clearly in Figure 4a and b that the Te s states match up with p states in the CB bottom (0.2 eV). The electrons can easily enter the p orbits and therefore form the donor. This may be the key reason for the ZnSb exhibiting *n*-type conductivity. Interestingly, for an S3 system as shown in Figure 4c, the ZnSb belongs to the *p*-type conductivity, the Te doped lead to a deep state in VB top, and the acceptor state is attributed by the Sb p states, which can contribute to the *p*-type conductivity.

These findings suggest that 1.56at% and 2.34at% are eminently suitable for donor dopant in the fabrication of *n*-type ZnSb crystals, while a 3.12at% doping amount of Te can contribute to the *p*-type conductivity for ZnSb, which is in good agreement with previous experimental study [8]. So, the Te concentration may severely affect the intrinsic properties of ZnSb, although further experiments are required to confirm this speculation. Technologically, making use of this simulation mechanism and amplifying the supercell model to simulate the different concentration as well as different type of dopant, we can understand the doping effect on the electronic structure and properties of a ZnSb system.

## 4. Conclusions

To conclude, the atomistic calculation on a Zn_64_Sb_64−_*_x_*Te*_x_* system shows that Te doping will lead to the ZnSb transfer from *p*-type to *n*-type conductivity. In the cases of Zn_64_Sb_61_Te_2_and Zn_64_Sb_60_Te_3_ systems, *n*-type behavior is primarily due to s and p states of the Te atom, which consequently provides a weak but visible peak as the donors in CB bottom. This simulation mechanism is not necessarily limited to the case of ZnSb, but may also apply to other types of ZnTe or CdTe compounds. Technolocally, the individual *n*- and *p*-type may hold the potential for the discovery of unusual application to functional materials.

## Figures and Tables

**Table 1.** Theoretical results and experimental data of lattice parameters.

	**a (Å)**	**b (Å)**	**c (Å)**
Calculation value in this study	6.216	7.784	8.231
Experimental data [18]	6.220	7.742	8.120
Calculation value in reference [12]	6.214	7.857	8.304
